# Hysteresis Modelling and Feedforward Control of Piezoelectric Actuator Based on Simplified Interval Type-2 Fuzzy System

**DOI:** 10.3390/s20092587

**Published:** 2020-05-02

**Authors:** Peng-Zhi Li, De-Fu Zhang, Jun-Yan Hu, Barry Lennox, Farshad Arvin

**Affiliations:** 1 Robotics for Extreme Environments Lab, Department of Electrical and Electronic Engineering, University of Manchester, Manchester M13 9PL, UK; junyan.hu@manchester.ac.uk (J.-Y.H.); barry.lennox@manchester.ac.uk (B.L.); 2 Changchun Institute of Optics, Fine Mechanics and Physics, Chinese Academy of Sciences, Changchun 130033, China; zhangdf@sklao.ac.cn

**Keywords:** hysteresis, piezoelectric actuator, interval type-2 fuzzy system, feedforward control, gradient based optimization

## Abstract

The piezoelectric actuator is indispensable for driving the micro-manipulator. In this paper, a simplified interval type-2 (IT2) fuzzy system is proposed for hysteresis modelling and feedforward control of a piezoelectric actuator. The partial derivative of the output of IT2 fuzzy system with respect to the modelling parameters can be analytically computed with the antecedent part of IT2 fuzzy rule specifically designed. In the experiments, gradient based optimization was used to identify the IT2 fuzzy hysteresis model. Results showed that the maximum error of model identification is 0.42% with only 3 developed IT2 fuzzy rules. Moreover, the model validation was conducted to demonstrate the generalization performance of the identified model. Based on the analytic inverse of the developed model, feedforward control experiment for tracking sinusoidal trajectory of 20 Hz was carried out. As a result, the hysteresis effect of the piezoelectric actuator was reduced with the maximum tracking error being 4.6%. Experimental results indicated an improved performance of the proposed IT2 fuzzy system for hysteresis modelling and feedforward control of the piezoelectric actuator.

## 1. Introduction

Smart material based actuators are new types of actuators different from the traditional electromagnetic actuators. Among these smart actuators, the piezoelectric actuator is widely used in the field of micro-/nano- positioning and manipulation [1,2,3,4,5,6], biomedical robotics and extreme environments [7,8,9,10] and optics [11,12,13] due to its extraordinary characteristics such as nanometer-scale displacement resolution, nonexistent friction and fast response.

However, the nonlinear hysteresis of the piezoelectric actuator has an influence on the positioning or manipulating accuracy of these applications. Under the hysteresis effect, the displacement of the piezoelectric actuator is a function of not only the current input voltage but also the previous displacement or input voltage.

Various modelling methods and control strategies have been proposed to tackle the hysteresis and its effect. The Prandtl-Ishlinskii model [14,15,16,17] was widely investigated for describing the rate-independent and rate-dependent, symmetric and asymmetric hysteresis. Preisach model [18,19], Duhem model [20], fuzzy system [21,22] and neural networks [23] were also presented to characterizing hysteresis. Regarding control strategies, feedback control algorithms incorporating feedforward control were mainly developed, such as finite-time learning control [24], iterative control [25,26], internal model-based feedback control [17] and fuzzy control [27].

Extensive research works into interval type-2 (IT2) fuzzy system were carried out during the last decades [28]. In fact, IT2 fuzzy system has more freedom of flexibility to describe the complex phenomenon than traditional type-1 fuzzy system, that makes it capable of modelling the nonlinear system more precisely. It has been applied in nonlinear modelling and automatic control in various studies [29,30,31,32,33,34].

In this paper, an IT2 fuzzy system with an analytic inverse was designed, and applied to hysteresis modelling and feedforward control of the stacked piezoelectric actuator with practical experiments. The remaining organization of this paper is as follows: in Section 2 the simplified IT2 fuzzy system is developed, Section 3 includes the experimental results, and Section 4 concludes the paper.

## 2. Simplified Interval Type-2 Fuzzy System

### 2.1. Basic Concepts

A type-1 fuzzy set *A* is a set function on the universe *X* into [0,1] [28], and a type-1 membership function (MF) of the type-1 fuzzy set *A* is denoted as $\mu_{A}\left(x\right)$, i.e.

(1) $$A=\left\{\left(x,\mu_{A}\left(x\right)\right)|x\in X,\;0\leq \mu_{A}\left(x\right)\leq 1\right\},$$

where *X* provides the allowable values for the variable *x*.

The support of a type-1 fuzzy set *A* is the crisp set of all the following points

(2) $$\left\{x|x\in X,\;\mu_{A}\left(x\right)>0\right\}.$$

A type-2 fuzzy set $\tilde{A}$ is the graph of a bivariate function on the Cartesian product X×[0,1] into [0,1], and a type-2 membership function of the type-2 fuzzy set $\tilde{A}$ is denoted as $\mu_{\tilde{A}}\left(x,u\right)$, i.e.

(3) $$\tilde{A}=\left\{\left(\left(x,u\right),\mu_{\tilde{A}}\left(x,u\right)\right)|x\in X,u\in U\equiv \left[0,1\right],\;0\leq \mu_{\tilde{A}}\left(x,u\right)\leq 1\right\},$$

where *X* and *U* are the universes for the primary variable *x* and the secondary variable *u*, respectively.

The footprint of uncertainty (FOU) of the type-2 fuzzy set $\tilde{A}$ is defined as

(4) $$\mathrm{FOU}\left(\tilde{A}\right)=\left\{\left(x,u\right)|x\in X,u\in \left[{\underline{\mu }}_{\tilde{A}}\left(x\right),{\bar{\mu }}_{\tilde{A}}\left(x\right)\right]\right\},$$

where ${\underline{\mu }}_{\tilde{A}}\left(x\right)$ and ${\bar{\mu }}_{\tilde{A}}\left(x\right)$ are the lower membership function (LMF) and upper membership function (UMF) of $\mathrm{FOU}(\tilde{A})$ respectively in the following forms

(5) $${\underline{\mu }}_{\tilde{A}}\left(x\right)=\inf\left\{u|u\in \left[0,1\right],\mu_{\tilde{A}}\left(x,u\right)>0\right\},$$

(6) $${\bar{\mu }}_{\tilde{A}}\left(x\right)=\sup\left\{u|u\in \left[0,1\right],\mu_{\tilde{A}}\left(x,u\right)>0\right\}.$$

The type-2 fuzzy set $\tilde{A}$ becomes an interval type-2 (IT2) fuzzy set when u∈[0,1] and $\mu_{\tilde{A}}\left(x,u\right)=1$ for x∈X.

### 2.2. Model

The simplified IT2 fuzzy system has the following $l^{\mathrm{th}}$ Takagi-Sugeno (T-S) [35] fuzzy rules:

(7) $${\tilde{R}}^{l}:\;\mathrm{IF}\;y\left(k-1\right)\;\mathrm{is}\;{\tilde{A}}^{l},\;\mathrm{THEN}\;y\left(k\right)=q_{l1}y\left(k-1\right)+q_{l2}x\left(k\right)+q_{l3},\;l=1,\cdots ,L$$

where $y\left(k\right)=y\left(kT_{s}\right)=y_{k},x\left(k\right)=x\left(kT_{s}\right)=x_{k}$ are the discrete time output and input of the modelled plant with hysteresis at the time instant $kT_{s}$, respectively. $T_{s}$ is the sampling period, $q_{l1},q_{l2},q_{l3}$ are the crisp parameters of the consequent part (i.e. THEN part of the fuzzy rule), and *L* is the number of fuzzy rules.

${\tilde{A}}^{l}$ in the antecedent part (i.e. IF part of the fuzzy rule) is a IT2 fuzzy set obtained by blurring the standard deviation of a Gaussian type-1 fuzzy set. The LMF and UMF of the FOU of ${\tilde{A}}^{l}$ are respectively

(8) $${\underline{\mu }}_{{\tilde{A}}^{l}}^{l}\left(y_{k-1}\right)=\exp\left(-\frac{{\left(y_{k-1}-c_{l}\right)}^{2}}{2\sigma_{l1}^{2}}\right),$$

(9) $${\bar{\mu }}_{{\tilde{A}}^{l}}^{l}\left(y_{k-1}\right)=\exp\left(-\frac{{\left(y_{k-1}-c_{l}\right)}^{2}}{2\sigma_{l2}^{2}}\right),$$

where $c_{l},\sigma_{l1},\sigma_{l2}$ are the crisp parameters of ${\tilde{A}}^{l}$.

The final defuzzified output of the IT2 fuzzy system can be determined by

(10) $${\hat{y}}_{k}=\frac{\sum_{l=1}^{L}\left(\left(q_{l1}y_{k-1}+q_{l2}x_{k}+q_{l3}\right)\left({\underline{f}}_{k}^{l}+{\bar{f}}_{k}^{l}\right)\right)}{\sum_{l=1}^{L}\left({\underline{f}}_{k}^{l}+{\bar{f}}_{k}^{l}\right)},$$

where ${\underline{f}}_{k}^{l}$ and ${\bar{f}}_{k}^{l}$ are the firing interval value of the $l^{\mathrm{th}}$ IT2 fuzzy rule in the following forms respectively

(11) $${\underline{f}}_{k}^{l}={\underline{\mu }}_{{\tilde{A}}^{l}}^{l}\left(y_{k-1}\right),$$

(12) $${\bar{f}}_{k}^{l}={\bar{\mu }}_{{\tilde{A}}^{l}}^{l}\left(y_{k-1}\right).$$

Some remarks regarding the proposed simplified design of the developed IT2 fuzzy system are as follows:

- The fuzzy system uses singleton fuzzifier and direct defuzzifier. Without type-reduction, the output of the fuzzy system is analytically computed via the Nie-Tan method [36]. It can reduce the computational burden without much loss of performance compared with the iterative Karnik-Mendal method [37]. Besides, it is feasible to derive the analytic gradient of the output function in (10) of the modelling parameters $c_{l},\sigma_{l1},\sigma_{l2},q_{l1},q_{l2},$ and $q_{l3}$, which gives much convenience of using gradient based optimization method. Moreover, due to the computational simplicity, the proposed IT2 fuzzy system can be practically applied to the open-loop feedforward controller for compensating the hysteresis effect.
- There are 2 variables, $y_{k-1}$ and $x_{k}$, in the consequent part of the fuzzy rule whilst only 1 variable $y_{k-1}$ in the antecedent part. This design is vital for obtaining the analytic inverse of the fuzzy system without $x_{k}$ in the antecedent part of the fuzzy rule. In fact, the proposed fuzzy rule in (7) is the same as the IT2 fuzzy rule:
  ${\tilde{R}}^{l}:\;IF\;y\left(k-1\right)\;\mathrm{is}\;{\tilde{A}}^{l}\;\mathrm{and}\;x\left(k\right)\;\mathrm{is}\;{\tilde{A}}_{x}^{l},\;\mathrm{THEN}\;y\left(k\right)=q_{l1}y\left(k-1\right)+q_{l2}x\left(k\right)+q_{l3}$ where ${\tilde{A}}_{x}^{l}$ is a IT2 fuzzy set whose LMF and UMF are constantly equal to 1, i.e., ${\underline{\mu }}_{{\tilde{A}}_{x}^{l}}^{l}\left(x_{k}\right)={\bar{\mu }}_{{\tilde{A}}_{x}^{l}}^{l}\left(x_{k}\right)=1$. This design also simplifies the identification of the modelling parameters and the computation of their partial derivative.

### 2.3. Optimization

The analytic partial derivative of the output function in (10) with respect to the modelling parameters $c_{l},\sigma_{l1},\sigma_{l2}$, $q_{l1},q_{l2},$ and $q_{l3}$ are:

(13) $$\begin{array}{cc} \frac{\partial {\hat{y}}_{k}}{\partial c_{l}} & =\left({\underline{f}}_{k}^{l}\cdot \frac{y_{k-1}-c_{l}}{{\sigma_{l1}}^{2}}+{\bar{f}}_{k}^{l}\cdot \frac{y_{k-1}-c_{l}}{{\sigma_{l2}}^{2}}\right)\cdot \{\frac{q_{l1}y_{k-1}+q_{l2}x_{k}+q_{l3}}{\sum_{l=1}^{L}\left({\underline{f}}_{k}^{l}+{\bar{f}}_{k}^{l}\right)}-\\ \; & \frac{\sum_{l=1}^{L}\left(\left(q_{l1}y_{k-1}+q_{l2}x_{k}+q_{l3}\right)\left({\underline{f}}_{k}^{l}+{\bar{f}}_{k}^{l}\right)\right)}{{\left(\sum_{l=1}^{L}\left({\underline{f}}_{k}^{l}+{\bar{f}}_{k}^{l}\right)\right)}^{2}}\} \end{array}$$

(14) $$\frac{\partial {\hat{y}}_{k}}{\partial \sigma_{l1}}={\underline{f}}_{k}^{l}\cdot \frac{{\left(y_{k-1}-c_{l}\right)}^{2}}{\sigma_{l1}^{3}}\cdot \left\{\frac{q_{l1}y_{k-1}+q_{l2}x_{k}+q_{l3}}{\sum_{l=1}^{L}\left({\underline{f}}_{k}^{l}+{\bar{f}}_{k}^{l}\right)}-\frac{\sum_{l=1}^{L}\left(\left(q_{l1}y_{k-1}+q_{l2}x_{k}+q_{l3}\right)\left({\underline{f}}_{k}^{l}+{\bar{f}}_{k}^{l}\right)\right)}{{\left(\sum_{l=1}^{L}\left({\underline{f}}_{k}^{l}+{\bar{f}}_{k}^{l}\right)\right)}^{2}}\right\}$$

(15) $$\frac{\partial {\hat{y}}_{k}}{\partial \sigma_{l2}}={\bar{f}}_{k}^{l}\cdot \frac{{\left(y_{k-1}-c_{l}\right)}^{2}}{\sigma_{l2}^{3}}\cdot \left\{\frac{q_{l1}y_{k-1}+q_{l2}x_{k}+q_{l3}}{\sum_{l=1}^{L}\left({\underline{f}}_{k}^{l}+{\bar{f}}_{k}^{l}\right)}-\frac{\sum_{l=1}^{L}\left(\left(q_{l1}y_{k-1}+q_{l2}x_{k}+q_{l3}\right)\left({\underline{f}}_{k}^{l}+{\bar{f}}_{k}^{l}\right)\right)}{{\left(\sum_{l=1}^{L}\left({\underline{f}}_{k}^{l}+{\bar{f}}_{k}^{l}\right)\right)}^{2}}\right\}$$

(16) $$\frac{\partial {\hat{y}}_{k}}{\partial q_{l1}}=\frac{y_{k-1}\left({\underline{f}}_{k}^{l}+{\bar{f}}_{k}^{l}\right)}{\sum_{l=1}^{L}\left({\underline{f}}_{k}^{l}+{\bar{f}}_{k}^{l}\right)}$$

(17) $$\frac{\partial {\hat{y}}_{k}}{\partial q_{l2}}=\frac{x_{k}\left({\underline{f}}_{k}^{l}+{\bar{f}}_{k}^{l}\right)}{\sum_{l=1}^{L}\left({\underline{f}}_{k}^{l}+{\bar{f}}_{k}^{l}\right)}$$

(18) $$\frac{\partial {\hat{y}}_{k}}{\partial q_{l3}}=\frac{{\underline{f}}_{k}^{l}+{\bar{f}}_{k}^{l}}{\sum_{l=1}^{L}\left({\underline{f}}_{k}^{l}+{\bar{f}}_{k}^{l}\right)}$$

The partial derivative of the output function in (10) with respect to the modelling parameter $c_{l}$ is

(19) $$\begin{array}{cc} \frac{\partial {\hat{y}}_{k}}{\partial c_{l}} & =\frac{\frac{\partial \left(\sum_{l=1}^{L}\left(\left(q_{l1}y_{k-1}+q_{l2}x_{k}+q_{l3}\right)\left({\underline{f}}_{k}^{l}+{\bar{f}}_{k}^{l}\right)\right)\right)}{\partial c_{l}}}{\sum_{l=1}^{L}\left({\underline{f}}_{k}^{l}+{\bar{f}}_{k}^{l}\right)}-\frac{\left(\sum_{l=1}^{L}\left(\left(q_{l1}y_{k-1}+q_{l2}x_{k}+q_{l3}\right)\left({\underline{f}}_{k}^{l}+{\bar{f}}_{k}^{l}\right)\right)\right)\frac{\partial \left(\sum_{l=1}^{L}\left({\underline{f}}_{k}^{l}+{\bar{f}}_{k}^{l}\right)\right)}{\partial c_{l}}}{{\left(\sum_{l=1}^{L}\left({\underline{f}}_{k}^{l}+{\bar{f}}_{k}^{l}\right)\right)}^{2}}\\ \; & =\frac{\frac{\partial \left(\left(q_{l1}y_{k-1}+q_{l2}x_{k}+q_{l3}\right)\left({\underline{f}}_{k}^{l}+{\bar{f}}_{k}^{l}\right)\right)}{\partial c_{l}}}{\sum_{l=1}^{L}\left({\underline{f}}_{k}^{l}+{\bar{f}}_{k}^{l}\right)}-\frac{\left(\sum_{l=1}^{L}\left(\left(q_{l1}y_{k-1}+q_{l2}x_{k}+q_{l3}\right)\left({\underline{f}}_{k}^{l}+{\bar{f}}_{k}^{l}\right)\right)\right)\frac{\partial \left({\underline{f}}_{k}^{l}+{\bar{f}}_{k}^{l}\right)}{\partial c_{l}}}{{\left(\sum_{l=1}^{L}\left({\underline{f}}_{k}^{l}+{\bar{f}}_{k}^{l}\right)\right)}^{2}}\\ \; & =\frac{\left(q_{l1}y_{k-1}+q_{l2}x_{k}+q_{l3}\right)\frac{\partial \left({\underline{f}}_{k}^{l}+{\bar{f}}_{k}^{l}\right)}{\partial c_{l}}}{\sum_{l=1}^{L}\left({\underline{f}}_{k}^{l}+{\bar{f}}_{k}^{l}\right)}-\frac{\left(\sum_{l=1}^{L}\left(\left(q_{l1}y_{k-1}+q_{l2}x_{k}+q_{l3}\right)\left({\underline{f}}_{k}^{l}+{\bar{f}}_{k}^{l}\right)\right)\right)\frac{\partial \left({\underline{f}}_{k}^{l}+{\bar{f}}_{k}^{l}\right)}{\partial c_{l}}}{{\left(\sum_{l=1}^{L}\left({\underline{f}}_{k}^{l}+{\bar{f}}_{k}^{l}\right)\right)}^{2}}\\ \; & =\frac{\partial \left({\underline{f}}_{k}^{l}+{\bar{f}}_{k}^{l}\right)}{\partial c_{l}}\left\{\frac{q_{l1}y_{k-1}+q_{l2}x_{k}+q_{l3}}{\sum_{l=1}^{L}\left({\underline{f}}_{k}^{l}+{\bar{f}}_{k}^{l}\right)}-\frac{\sum_{l=1}^{L}\left(\left(q_{l1}y_{k-1}+q_{l2}x_{k}+q_{l3}\right)\left({\underline{f}}_{k}^{l}+{\bar{f}}_{k}^{l}\right)\right)}{{\left(\sum_{l=1}^{L}\left({\underline{f}}_{k}^{l}+{\bar{f}}_{k}^{l}\right)\right)}^{2}}\right\}\\ \; & =\left({\underline{f}}_{k}^{l}\cdot \frac{y_{k-1}-c_{l}}{{\sigma_{l1}}^{2}}+{\bar{f}}_{k}^{l}\cdot \frac{y_{k-1}-c_{l}}{{\sigma_{l2}}^{2}}\right)\cdot \left\{\frac{q_{l1}y_{k-1}+q_{l2}x_{k}+q_{l3}}{\sum_{l=1}^{L}\left({\underline{f}}_{k}^{l}+{\bar{f}}_{k}^{l}\right)}-\frac{\sum_{l=1}^{L}\left(\left(q_{l1}y_{k-1}+q_{l2}x_{k}+q_{l3}\right)\left({\underline{f}}_{k}^{l}+{\bar{f}}_{k}^{l}\right)\right)}{{\left(\sum_{l=1}^{L}\left({\underline{f}}_{k}^{l}+{\bar{f}}_{k}^{l}\right)\right)}^{2}}\right\} \end{array}$$

The derivation of (14) and (15) are similar to the process described in (19) above, so their detailed deriving processes are omitted for brevity of this paper.

Therefore, the gradient of ${\hat{y}}_{k}$ in (10) with respect to the modelling parameters $p^{l}=\left(c_{l},\sigma_{l1},\sigma_{l2},q_{l1},q_{l2},q_{l3}\right)$ of the $l^{\mathrm{th}}$ IT2 fuzzy rule is

(20) $$\nabla {\hat{y}}_{k}\left(p^{l}\right)=\left(\frac{\partial {\hat{y}}_{k}}{\partial c_{l}},\frac{\partial {\hat{y}}_{k}}{\partial \sigma_{l1}},\frac{\partial {\hat{y}}_{k}}{\partial \sigma_{l2}},\frac{\partial {\hat{y}}_{k}}{\partial q_{l1}},\frac{\partial {\hat{y}}_{k}}{\partial q_{l2}},\frac{\partial {\hat{y}}_{k}}{\partial q_{l3}}\right).$$

Based on the gradient in (20), many gradient based optimization methods can be used. The basic gradient descent method iteratively updates the optimized parameters such that

(21) $$p_{i+1}=p_{i}-\gamma_{i}\nabla {\hat{y}}_{k}\left(p_{i}\right),$$

where $p_{i}=\left(p_{i}^{1},\cdots ,p_{i}^{l},\cdots ,p_{i}^{L}\right)$ denotes all the modelling parameters of the IT2 fuzzy system to be optimized in the $i^{\mathrm{th}}$ iteration and $\gamma_{i}>0$ is called the step size which is allowed to change at every iteration.

## 3. Experiments

### 3.1. Experimental Platform

**Figure 1:**
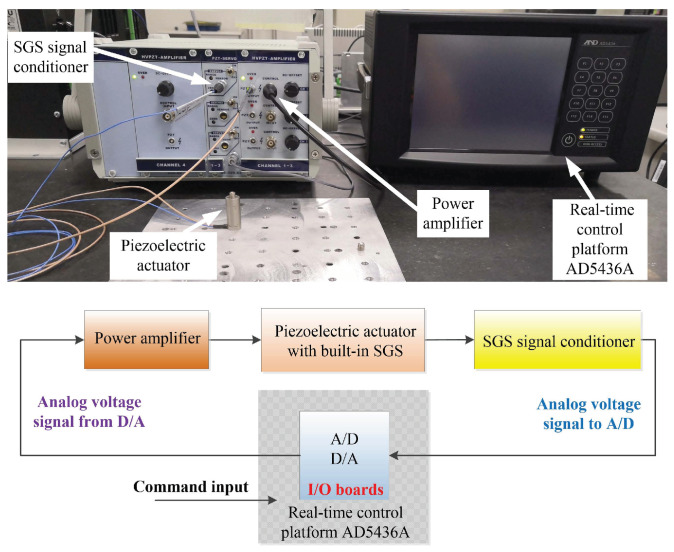
Experimental platform: (**top**) main hardware including piezoelectric actuator, power amplifier, strain gauge sensor signal conditioner, and real-time control platform AD5436A, (**bottom**) signal flow.

The experimental platform is mainly comprised of 4 parts: (1) piezoelectric actuator; (2) power amplifier; (3) strain gauge sensor (SGS) signal conditioner, and (4) real-time control platform (RTCP) AD5436A, as shown in Figure 1 (top). The stacked piezoelectric actuator 20VS12 to be modelled and feedforward controlled has a built-in SGS to monitor its displacement. The nominal travel range of the piezoelectric actuator is 16 μm and the piezoelectric actuator is fixed on an optical vibration isolation platform for rejecting external vibrational disturbance. The power amplifier XE-503.00 can amplify the input 0∼10 V analog voltage by 15 times and output 0∼150 V analog voltage with an average power of 7 W to excite the piezoelectric actuator. The SGS signal conditioner XE-509.S3 converts the signal generated from SGS into 0∼10 V analog voltage with a 0.1% nonlinearity. The piezoelectric actuator, power amplifier and SGS signal conditioner are all manufactured by Harbin Core Tomorrow Science and Technology Co., Ltd. in China. The RTCP AD5436A consists of an Intel Core i7-610E 2.53 GHz dual-Core CPU, 16 bits A/D and 16 bits D/A converter I/O boards. It is used for high-speed measurement of the displacement and rapid control prototyping for feedforward control of the piezoelectric actuator. As illustrated in Figure 1 (bottom), the experimental schematic diagram shows the signal flow of the main components of the experimental platform.

### 3.2. Model Identification

**Figure 2:**
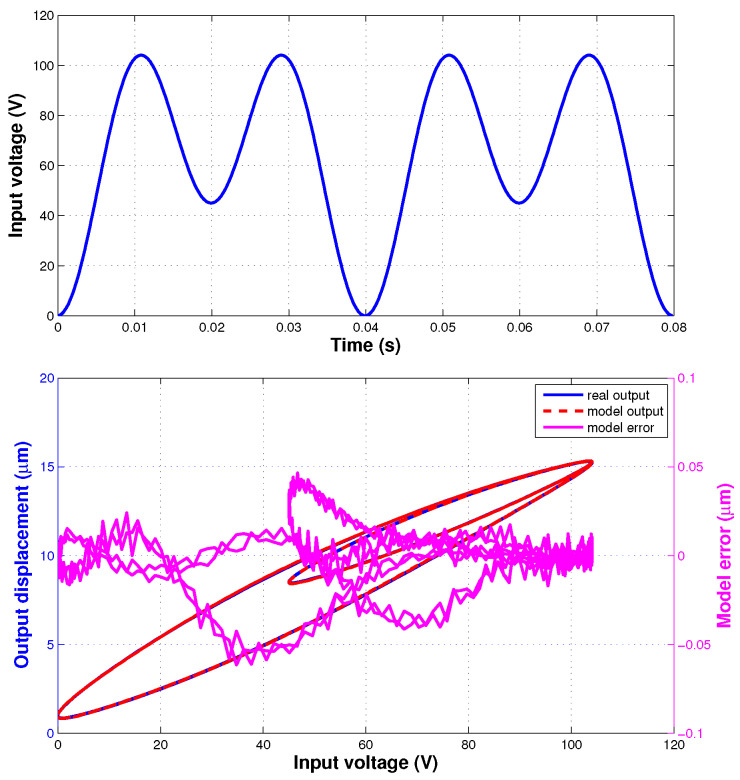
Model Identification: (**top**) input voltage signal, (**bottom**) identification result and error.

As shown in Figure 2 (top), the input voltage signal used for the hysteresis model identification of the IT2 fuzzy system is described with the following function

(22) $$x\left(k\right)=40\sin\left(2\pi \;\cdot \;50\;\cdot \;kT_{s}-\frac{\pi }{2}\right)+22.5\sin\left(2\pi \;\cdot \;25\;\cdot \;kT_{s}-\frac{\pi }{2}\right)+62.5,$$

where $T_{s}=0.0001$ s means that the sampling frequency is 10 kHz. The signal is the sum of 2 different sinusoidal profiles with 50 Hz and 25 Hz frequencies.

The cost function for optimization is defined as

(23) $$\frac{1}{2}\sum_{k=1}^{N}{\left(y\left(k\right)-{\hat{y}}_{k}\right)}^{2},$$

where *N* is the number of the sampled data. The input x(k) and output y(k) of the piezoelectric actuator are extended as a vector $\left(\left(y(k-1),x(k)\right),y\left(k\right)\right)$. The vector is then used for the IT2 fuzzy system to model the hysteresis.

Hysteresis model identification of the proposed IT2 fuzzy system can be executed as follows:

1. Choose the value L=3 for the number of IT2 fuzzy rules.
2. Use gradient based method to optimize the antecedent and consequent parameters of IT2 fuzzy system based on (13−18). Gradient descent of (21) can be adopted or other similar methods such as Matlab function *fminunc* can also be used.
3. The IT2 fuzzy system of (7) is identified with the optimized parameters.
4. Use (10) to compute the output ${\hat{y}}_{k}$ of the hysteresis model.

**Figure 3:**
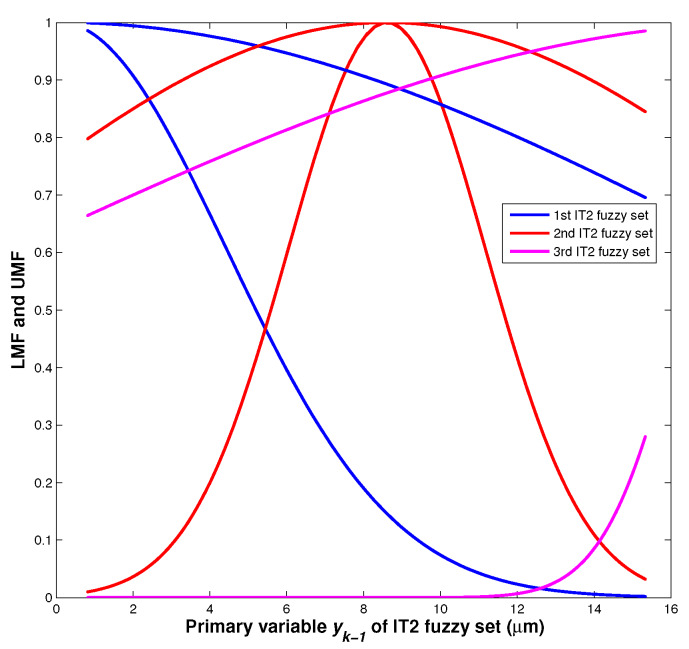
LMF and UMF of the identified IT2 fuzzy sets.

The identified parameters of IT2 fuzzy system are listed in Table 1. The LMF and UMF of the identified IT2 fuzzy sets are shown in Figure 3.

To evaluate the modelling performance, 2 types of error index are defined as:

(24) $$e_{mr}=\frac{\max\left(|y\left(k\right)-{\hat{y}}_{k}|\right)}{\max\left(y(k)\right)-\min\left(y(k)\right)}\times 100\%\;,$$

(25) $$e_{rms}=\sqrt{\frac{1}{N}\left(\sum_{k=1}^{N}{\left(y\left(k\right)-{\hat{y}}_{k}\right)}^{2}\right)}\;.$$

Figure 2(bottom) presents the identification result of the proposed hysteresis model. The $e_{mr}$ and $e_{rms}$ are 0.42% and 0.016 μm, respectively.

### 3.3. Model Validation

To validate the identified model, the following input voltage signals of different profiles are used:

(26) $$x_{ms}\left(k\right)=52.5\sin\left(2\pi \;\cdot \;f_{ms}\;\cdot \;kT_{s}-\frac{\pi }{2}\right)+52.5,$$

(27) $$x_{mt}\left(k\right)=x_{mt}\left(k+\frac{m}{f_{mt}T_{s}}\right)=\left\{\begin{array}{cc} 210\;\cdot \;f_{mt}\;\cdot \;kT_{s},\; & k\in [0,\frac{1}{2f_{mt}T_{s}})\\ -210\;\cdot \;f_{mt}\;\cdot \;kT_{s}+210,\; & k\in (\frac{1}{2f_{mt}T_{s}},\frac{1}{f_{mt}T_{s}}] \end{array}\right.,$$

where $f_{ms}$ and $f_{mt}$ are the frequencies of the sinusoidal and triangular signals, respectively, and $m\in \left\{0,1,2,\cdots \right\}$ is the period number of the triangular signal.

**Figure 4:**
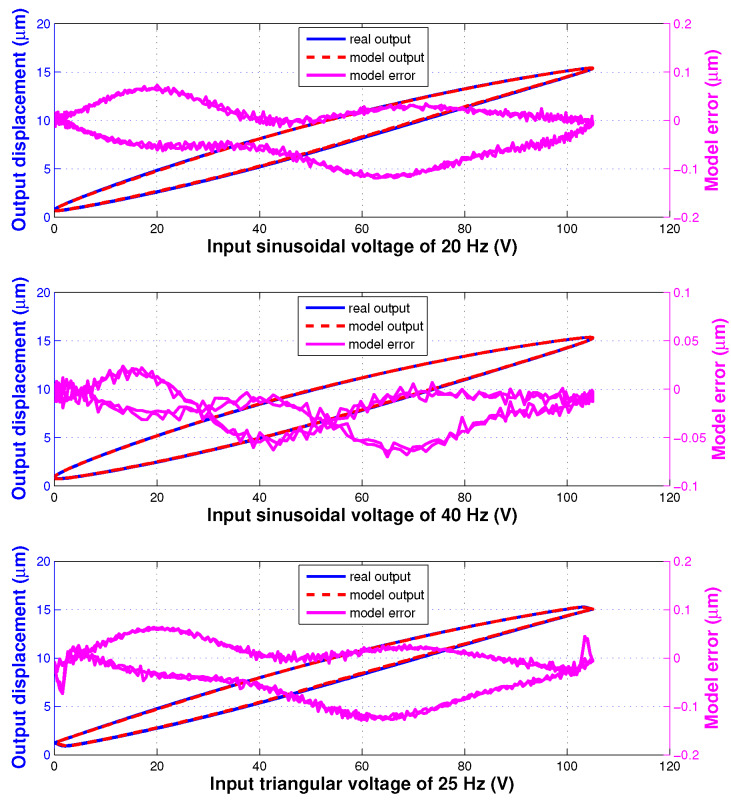
Validation results of the identified hysteresis model under different input signals: (**top**) 20 Hz sinusoidal, (**center**) 40 Hz sinusoidal, and (**bottom**) 25 Hz triangular.

**Figure 5:**
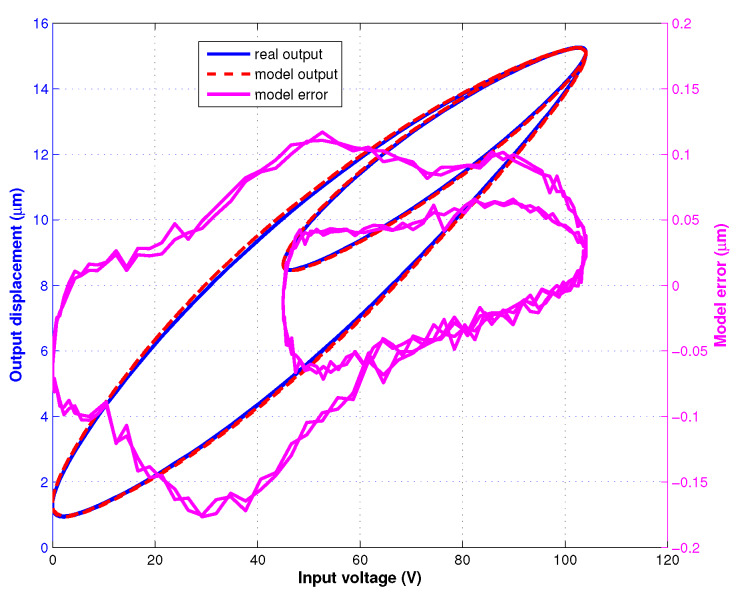
Validation results of the identified hysteresis model under the signal which is the sum of 2 different sinusoidal profiles with 100 Hz and 50 Hz frequencies.

In the model validation experiments, totally 4 different input signals were used to excite the piezoelectric actuator and the corresponding displacement was measured. These input signals consist of two sinusoidal signals of (26) with $f_{ms}=20$ Hz or 40 Hz, a triangular signal of (27) with $f_{mt}=25$ Hz and a signal which is the sum of 2 different sinusoidal profiles with 100 Hz and 50 Hz frequencies. The validation results of the identified hysteresis model are shown in Figure 4 and Figure 5. The modelling errors are presented in Table 2. These results demonstrate the generalization performance of the developed IT2 fuzzy hysteresis model.

The hysteresis model is firstly identified based on the measured data of the piezoelectric actuator under the excitatory input voltage for identification of (22). Then, the generalization performance of the identified model (its identified parameters are listed in Table 1) is validated by using other different sampled data under the excitatory input voltages for validation of (26) and (27).

### 3.4. Feedforward Control

Compared with feedback control, feedforward control does not indispensably need the expensive sensor for its practical implementation. It is suitable for the applications where the sensor is not feasible or easy to be deployed for directly monitoring the plant or where the cost is a top priority and strictly limited. The plant’s model and especially its inverse are generally needed for the feedforward control.

Based on (10), the equation can be rewritten as

(28) $$\begin{array}{cc} {\hat{y}}_{k}\left(\sum_{l=1}^{L}\left({\underline{f}}_{k}^{l}+{\bar{f}}_{k}^{l}\right)\right) & =\sum_{l=1}^{L}\left(\left(q_{l1}y_{k-1}+q_{l2}x_{k}+q_{l3}\right)\left({\underline{f}}_{k}^{l}+{\bar{f}}_{k}^{l}\right)\right)\\ \; & =\sum_{l=1}^{L}\left(\left(q_{l1}y_{k-1}+q_{l3}\right)\left({\underline{f}}_{k}^{l}+{\bar{f}}_{k}^{l}\right)\right)+\sum_{l=1}^{L}\left(q_{l2}x_{k}\left({\underline{f}}_{k}^{l}+{\bar{f}}_{k}^{l}\right)\right). \end{array}$$

Then, it can be transposed as

(29) $$x_{k}\sum_{l=1}^{L}\left(q_{l2}\left({\underline{f}}_{k}^{l}+{\bar{f}}_{k}^{l}\right)\right)={\hat{y}}_{k}\left(\sum_{l=1}^{L}\left({\underline{f}}_{k}^{l}+{\bar{f}}_{k}^{l}\right)\right)-\sum_{l=1}^{L}\left(\left(q_{l1}y_{k-1}+q_{l3}\right)\left({\underline{f}}_{k}^{l}+{\bar{f}}_{k}^{l}\right)\right).$$

Hence, the analytic inverse, $x_{inv}$, of the proposed hysteresis model based on IT2 fuzzy system is

(30) $$x_{inv}\left(k\right)=\frac{y_{k}\left(\sum_{l=1}^{L}\left({\underline{f}}_{k}^{l}+{\bar{f}}_{k}^{l}\right)\right)-\sum_{l=1}^{L}\left(\left(q_{l1}y_{k-1}+q_{l3}\right)\left({\underline{f}}_{k}^{l}+{\bar{f}}_{k}^{l}\right)\right)}{\sum_{l=1}^{L}\left(q_{l2}\left({\underline{f}}_{k}^{l}+{\bar{f}}_{k}^{l}\right)\right)}.$$

**Figure 6:**
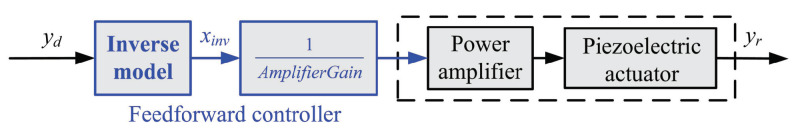
Block diagram of feedforward controller.

An open-loop feedforward controller is designed for the piezoelectric actuator based on the inverse model of (30) as shown in Figure 6. In the practical experiment, based on rapid control prototyping, this feedforward controller was implemented by the RTCP AD5436A under the real-time Xenomai operating system. The servo period was 0.1 ms, whose value is equal to the value of the sampling period $T_{s}$ of (22).

**Figure 7:**
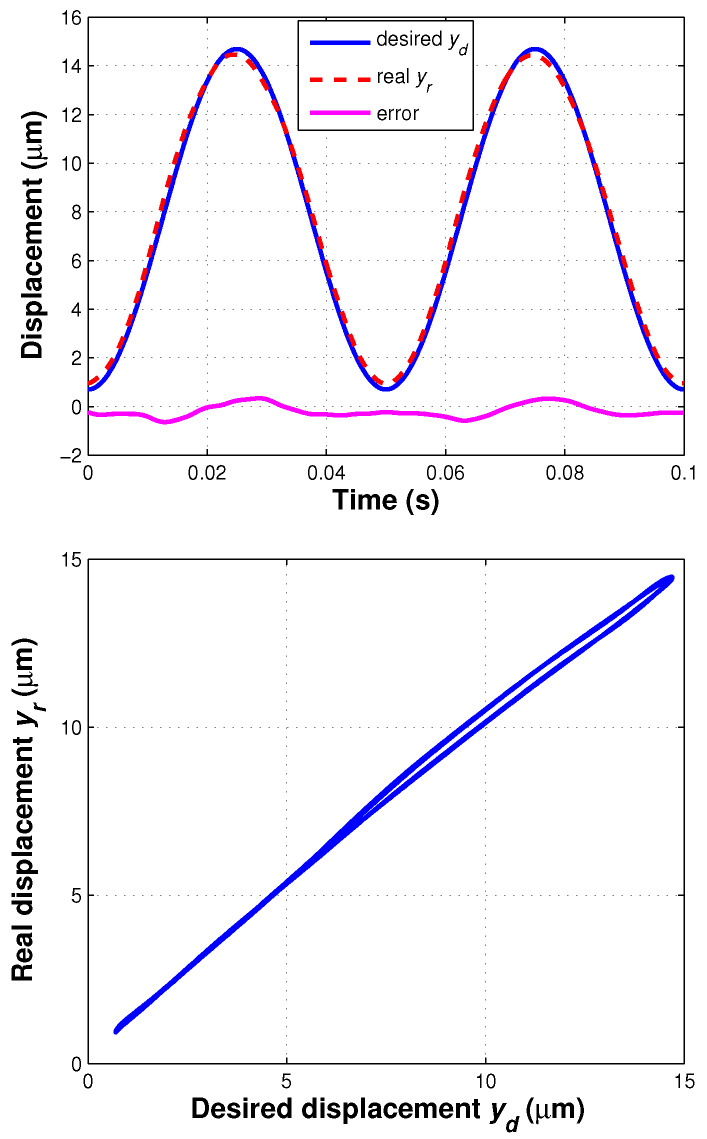
Tracking performance of feedforward controller: (**top**) tracking results, (**bottom**) hysteresis compensation result.

The desired displacement was chosen as $y_{d}\left(k\right)=7.0\sin(2\pi \;\cdot \;$$20\;\cdot \;kT_{s}-\pi /2)+7.7$ μm, whose frequency is 20 Hz. The tracking performance of the feedforward controller based on the inverse model of the developed IT2 fuzzy system is shown in Figure 7. The $e_{rms}$ and $e_{mr}$ of the sinusoidal trajectory tracking are 0.32 μm and 4.6%, respectively, and the hysteresis effect of the piezoelectric actuator was significantly compensated. When there is no such inverse model applied, $e_{mr}$ of a proportional feedforward controller can be 12.8%. Hence, the proposed feedforward controller has a good performance of tracking sinusoidal trajectory and compensating hysteresis of the piezoelectric actuator.

## 4. Conclusions

With analytic gradient and inverse, a simplified IT2 fuzzy system was developed for hysteresis modelling and feedforward control of the piezoelectric actuator. Experimental results demonstrated excellent performance of the proposed IT2 fuzzy system with only 3 fuzzy rules. Future work will involve: (1) other optimization methods such as evolutionary computation and neural networks for identifying the parameters of IT2 fuzzy system, and (2) feedback control algorithm incorporating the inverse model of IT2 fuzzy system.

## Figures and Tables

**Table 1:** Identified parameters of IT2 fuzzy system.

Rule	Antecedent Parameters	Consequent Parameters
1	(4.3427, 17.8879, 0.0892)	(0.8943, 0.0196, 1.7504)
2	(2.5647, 11.5798, 8.6067)	(0.9140, 0.0129, 0.8773)
3	(2.1349, 19.8162, 18.7381)	(1.1882, 0.0148, −6.2312)

**Table 2:** Output errors of the model validation.

Input Signal	$e_{\mathrm{mr}}$ (%)	$e_{\mathrm{rms}}$ (μm)
$f_{ms}=20$ Hz in (26)	0.81	0.047
$f_{ms}=40$ Hz in (26)	0.48	0.025
$f_{mt}=25$ Hz in (27)	0.90	0.055
sinusoidal 100 Hz + 50 Hz	1.23	0.067

## Abbreviations

The following abbreviations are used in this manuscript:

IT2	Interval type-2
MF	Membership function
FOU	Footprint of uncertainty
LMF	Lower membership function
UMF	Upper membership function
T-S	Takagi-Sugeno
SGS	Strain gauge sensor
RTCP	Real-time control platform
